# The Safety of Nebulized Conjugated Linoleic Acid for COVID-19 Respiratory Tract Infections

**DOI:** 10.7759/cureus.99733

**Published:** 2025-12-20

**Authors:** Sven T Jonsson, William J Beckworth

**Affiliations:** 1 Family Medicine, AdventHealth Hendersonville, Brevard, USA; 2 Pain Medicine, AdventHealth Hendersonville, Hendersonville, USA

**Keywords:** acute respiratory failure with hypoxia, antimicrobial resistance, antimicrobial therapy, breathing difficulty, chronic lung diseases, covid 19, lower respiratory airway, prevention of infections, respiratory viral infect, surfactant

## Abstract

Linoleic acid is an essential fatty acid, and in 2007, the US FDA listed conjugated linoleic acid (CLA) isomers as generally recognized as safe (GRAS) for human consumption. The potassium salt of conjugated linoleic acid (K-CLA) exhibits broad-spectrum antimicrobial activity. K-CLA also possesses mucolytic properties. This study reviewed the safety of nebulized K-CLA for COVID-19-related respiratory tract infections early during the pandemic. Among 57 treated subjects, adverse events leading to treatment discontinuation occurred in three patients. Two reported an increased cough, and the other reported nausea with emesis. Of the 57 patients treated, 25 were hypoxic during their infection, and 18 of the 25 reported benefits with dyspnea and/or pulse oximetry after initiation of nebulized K-CLA. This retrospective case series provides initial human safety data and describes observed clinical outcomes in subjects treated with nebulized K-CLA, supporting the need for prospective controlled trials.

## Introduction

Linoleic acid (LA) is an essential fatty acid obtained from both plant and animal dietary sources. Conjugated linoleic acid (CLA) refers to a family of geometric and positional isomers of LA composed of 18-carbon fatty acids with two conjugated double bonds. Notably, the cis-9, trans-11, and the trans-10, cis-12 isomers are the most commonly studied isomers [1-2]. In 2007, the FDA listed CLA isomers as generally recognized as safe (GRAS) for human use [3]. 

There have been a number of studies suggesting various forms of CLA have antiviral, antifungal, and antibacterial properties, although clinical translation remains uncertain [4-10]. Additionally, the SARS-CoV-2 spike protein has binding pockets that bind linoleic acid. This binding locks the spike protein into a more stable shape that reduces its ability to bind to the angiotensin-converting enzyme 2 (ACE2) receptor [11-12]. Beyond the spike protein associated with COVID-19, CLA has been reported to have broad antiviral, antibacterial, and even antifungal properties [4-10].

Yet CLA represents a mixture of isomers rather than a single compound, and results from studies involving specific formulations should not be generalized to all CLA products. The potassium salt of conjugated linoleic acid (K-CLA) has in vitro broad-spectrum antimicrobial effects on gram-positive bacteria, gram-negative bacteria, viruses, and fungi. In vitro testing performed at the Institute of Antiviral Research (Utah State) has demonstrated K-CLA kill-rates against SARS-CoV-2 on par with those of 63% ethanol. Specifically, in vitro testing of K-CLA 4.4-50.2 mM showed a 99% kill-rate of COVID-19 in two minutes [13]. Laboratory testing reported high viral reduction in vitro, though clinical relevance remains unknown.

Previous case reports have described the use of nebulized K-CLA in COVID-19, suggesting it is well-tolerated and may be associated with clinical improvement. It was speculated that the benefits may come from the antimicrobial effects of this specific K-CLA, and there may be benefits from the surfactant/mucolytic actions of K-CLA on respiratory infections [13].

The purpose of this study is to review the safety profile of nebulized K-CLA in the treatment of COVID-19 respiratory tract infections. While there have been some case reports previously, the goal of this study is to collect a larger data set on safety and report patient outcomes.

## Materials and methods

This was a retrospective chart review looking at all patients treated with nebulized K-CLA for respiratory tract infections with COVID-19 from October 2020 to August 2021. All patients aged 18 and older presenting during the study period with COVID-19-related medical concerns were offered treatment. The electronic medical record (EMR) was reviewed for all patients who were treated with nebulized K-CLA. Additionally, a logbook was reviewed that was kept at the time of treatments, which tracked how patients did. This logbook came from clinical visits and phone calls. Subjects gave verbal consent and were made aware that this was an off-label treatment and reviewed potential risks, benefits, and alternatives. Nebulized K-CLA was used as a compassionate use during the early part of the pandemic when there were not many other options. This was not an industry-sponsored study without funding.

Patients who received treatment were either symptomatic from COVID-19, asymptomatic COVID-19 (tested positive), significant COVID-19 exposure (without symptoms), or post-COVID-19 syndrome subjects. These patients were closely monitored by phone calls, follow-up, and some in-home visits by the clinician for side effects and improvements. Side effects that were documented included the ability to tolerate treatments, worsening respiratory status, pulse oximetry, and any other reported side effects. Exclusion criteria included any patients with acute COVID-19 requiring hospitalization, as well as individuals with other acute respiratory infections not caused by SARS-CoV-2. The primary outcome for this study was the incidence of adverse events leading to treatment discontinuation. Secondary outcomes included any symptoms reported during or after each treatment session, such as worsened dyspnea, cough, nausea, or declines in pulse oximetry readings.

Treatment prescribed was 1.96 mg K-CLA commercial preparation sold as a nasal-oral wash, Quorum Sense Now (QSN, Ceela Natural, Kentucky) via nebulizer. Nebulization was performed using 0.56 ml QSN (11 mM solution K-CLA) with 0.56 ml distilled water, providing a 5.5 mM solution. Patients were instructed to nebulize using this dose every four hours around the clock for seven days and longer if patients perceived ongoing benefit. The post-COVID-19 syndrome patients were treated with 1.96 mg K-CLA four times a day for one week, then three times a day for one week, then two times a day for one week, then once a day for one week, and then discontinued. After identifying an increased cough associated with nebulization, patients were instructed to inhale through the nose and exhale through the mouth, which provided more comfort during treatments. During August 2021, following the more aggressive COVID-19 delta surge, treatment increased to every two hours of nebulization while awake and every four hours at night.

Patients were treated by a primary care clinic in western North Carolina. This primary care clinic is part of AdventHealth Hendersonville, which is affiliated with a hospital that is part of a larger network of hospitals throughout the United States. AdventHealth Institutional Review Board issued approval number 2277003-3 for this study. The Common Rule and Good Clinical Practice International Conference on Harmonization (ICH) Guidelines were followed. 

## Results

A total of 57 patients with COVID-19-related complaints were treated with nebulized K-CLA. The group included 27 men and 30 women, ranging in age from 18 to 89 years. Of these patients, 49 were treated for acute COVID-19 infection, four received prophylactic treatment following significant exposure, three were asymptomatic but had recently tested positive for COVID-19, and one was treated for post-COVID infection syndrome.

Among the 57 patients, 41 were overweight or obese, eight had non-insulin-dependent diabetes mellitus (NIDDM), two had insulin-dependent diabetes mellitus (IDDM), 18 had essential hypertension, three had coronary artery disease, three had asthma, one had end-stage chronic kidney disease, three had thrombophilia, six had hypothyroidism, two had monoclonal gammopathy of undetermined significance (MGUS), eight had anxiety and/or depression, and four had a history of cancer (Table 1).

**Table 1:** Demographic and clinical characteristics of patients treated with nebulized potassium-conjugated linoleic acid NIDDM - non-insulin-dependent diabetes mellitus; IDDM - insulin-dependent diabetes mellitus; MGUS - monoclonal gammopathy of undetermined significance

Characteristics	Value, n (%)
Total patients	57 (100%)
Sex	
Male	27 (47.4%)
Female	30 (52.6%)
Age range, years	18-89
Clinical status	
Acute COVID-19 infection	49 (86.0%)
Prophylaxis after exposure	4 (7.0%)
Asymptomatic COVID-positive	3 (5.3%)
Post-COVID syndrome (long COVID)	1 (1.8%)
Comorbidities and Risk Factors	
Overweight or obese	41 (71.9%)
Diabetes mellitus, non-insulin dependent (NIDDM)	8 (14.0%)
Diabetes mellitus, insulin-dependent (IDDM)	2 (3.5%)
Essential hypertension	18 (31.6%)
Coronary artery disease	3 (5.3%)
Asthma	3 (5.3%)
End-stage chronic kidney disease	1 (1.8%)
Antiphospholipid syndrome	2 (3.5%)
Factor V Leiden mutation	2 (3.5%)
Elevated homocysteine	1 (1.8%)
Hypothyroidism	3 (5.3%)
Monoclonal gammopathy of undetermined significance (MGUS)	2 (3.5%)
Anxiety and/or depression	8 (14.0%)
History of cancer	4 (7.0%)
Patients presenting with hypoxia	25 (43.9%)

Three patients discontinued treatment early. One discontinued during the first nebulization session due to vomiting. Another patient discontinued during the first session due to an increase in cough. The third patient discontinued on the second day, also due to a cough. No other patients reported ongoing or concerning symptoms attributable to nebulization. These patients were primary care patients who were followed up after their treatment. No additional adverse events or side effects of the K-CLA have been reported either in the short- or long-term. The first two treated patients were provided informed consent after being advised of the potential risks and benefits of nebulized K-CLA as an off-label treatment. The first patient, a male, initiated treatment two days after symptom onset, while his spouse began treatment after the first day of symptoms. Both individuals were categorized as high risk due to medical comorbidities and experienced mild hypoxia during their illness. All treatments were administered at home. Symptom resolution was observed by day six and day seven [13]. Their recovery was notably shorter than expected in high-risk patients during the early stages of the pandemic [14-15]. No adverse treatment effects were reported. Both patients described unexpected improvement after K-CLA nebulization treatment and reported improved breathing comfort, which was supported by improvement in pulse oximetry [13].

**Figure 1:**
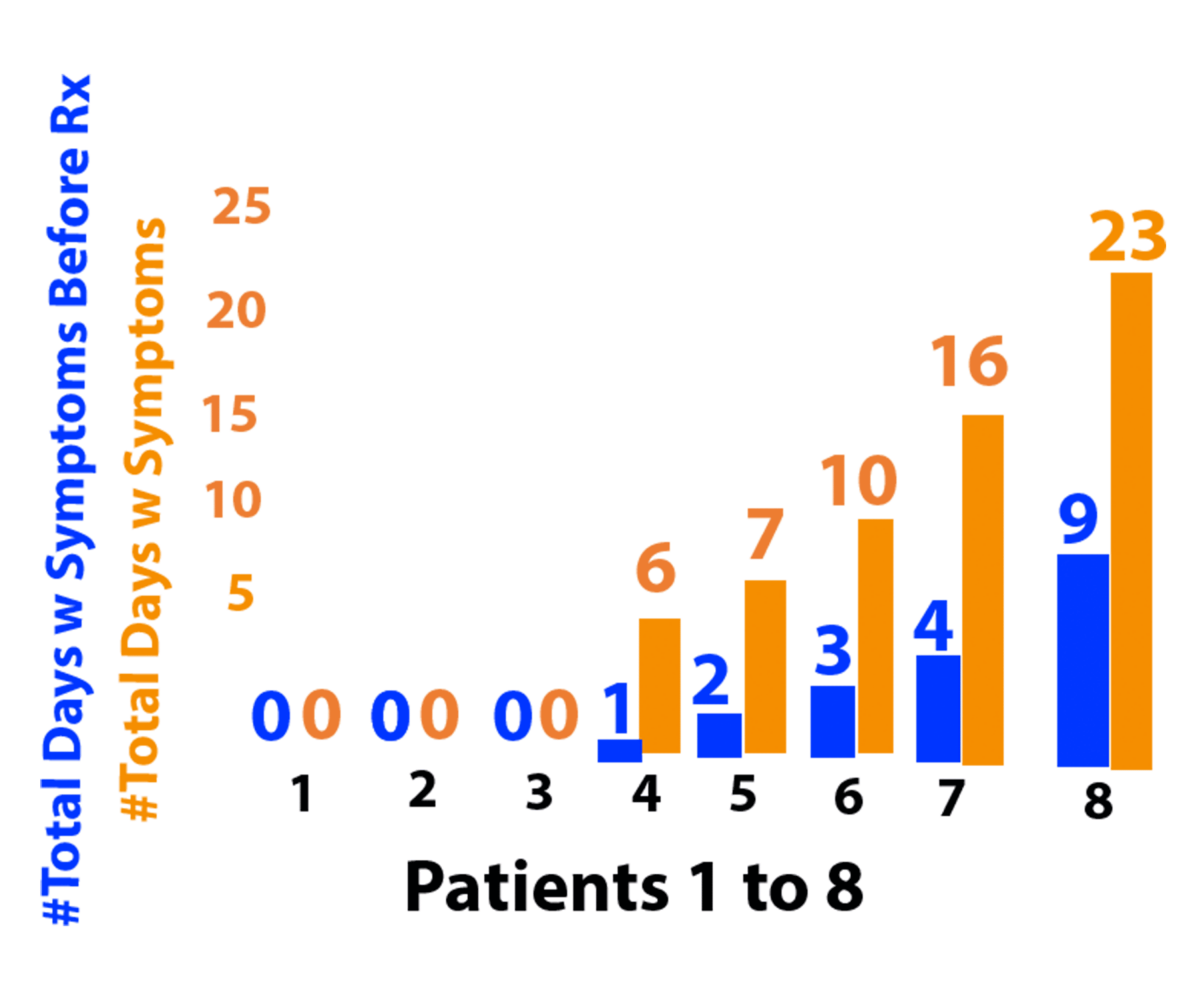
Early initiation of nebulized K-CLA therapy during COVID-19 infection was associated with shorter symptom duration Patients ranged in age from 56 to 74 years and included four males and four females. K-CLA - potassium salt of conjugated linoleic acid; Rx - treatment

After treating the first eight consecutive patients, an observational trend suggested that earlier initiation of therapy correlated with shorter patient-reported symptom duration (Figure 1). Based on the observed clinical improvement, nebulized K-CLA was subsequently offered to patients presenting with COVID-19.

As further patients with COVID-19-related hypoxia were treated, improvement in both breathing and oxygenation was reported by most subjects. The benefits of breathing and pulse-oximetry were typically evident soon after starting each treatment. Of the 49 patients treated for acute COVID-19, 25 were hypoxic during the course of their infection. Of these 25, 18 reported benefits in dyspnea and/or pulse oximetry after starting nebulized K-CLA. 

A representative case involved a patient with severe hypoxia secondary to COVID-19 pneumonia. He presented to his primary care physician, following multiple visits to the emergency room (ER) because of worsening hypoxemia. During his illness, this patient meticulously documented pulse oximetry readings and treatment details in a personal diary. Prior to his final ER visit, his oxygen saturation had dropped to 75% after temporarily removing supplemental oxygen during a bathroom visit. During his ER evaluation, his oxygen flow was increased up to three L/min, providing temporary pulse-oximetry benefit up to 90%. Due to a shortage of inpatient beds during a surge in COVID-19 cases, the patient was discharged back home. Later that day, he reported an increase in dyspnea, profound fatigue, and oxygen saturation of 85% to his primary care physician. He refused to return to the ER and requested nebulized K-CLA therapy. After providing informed consent for off-label use of nebulized K-CLA therapy, treatment was initiated. The patient experienced quick relief of dyspnea, reversal of hypoxia, and sustained improvement in oxygenation, allowing uninterrupted sleep between nebulization treatments, for the first time in over 48 hours. 

**Figure 2:**
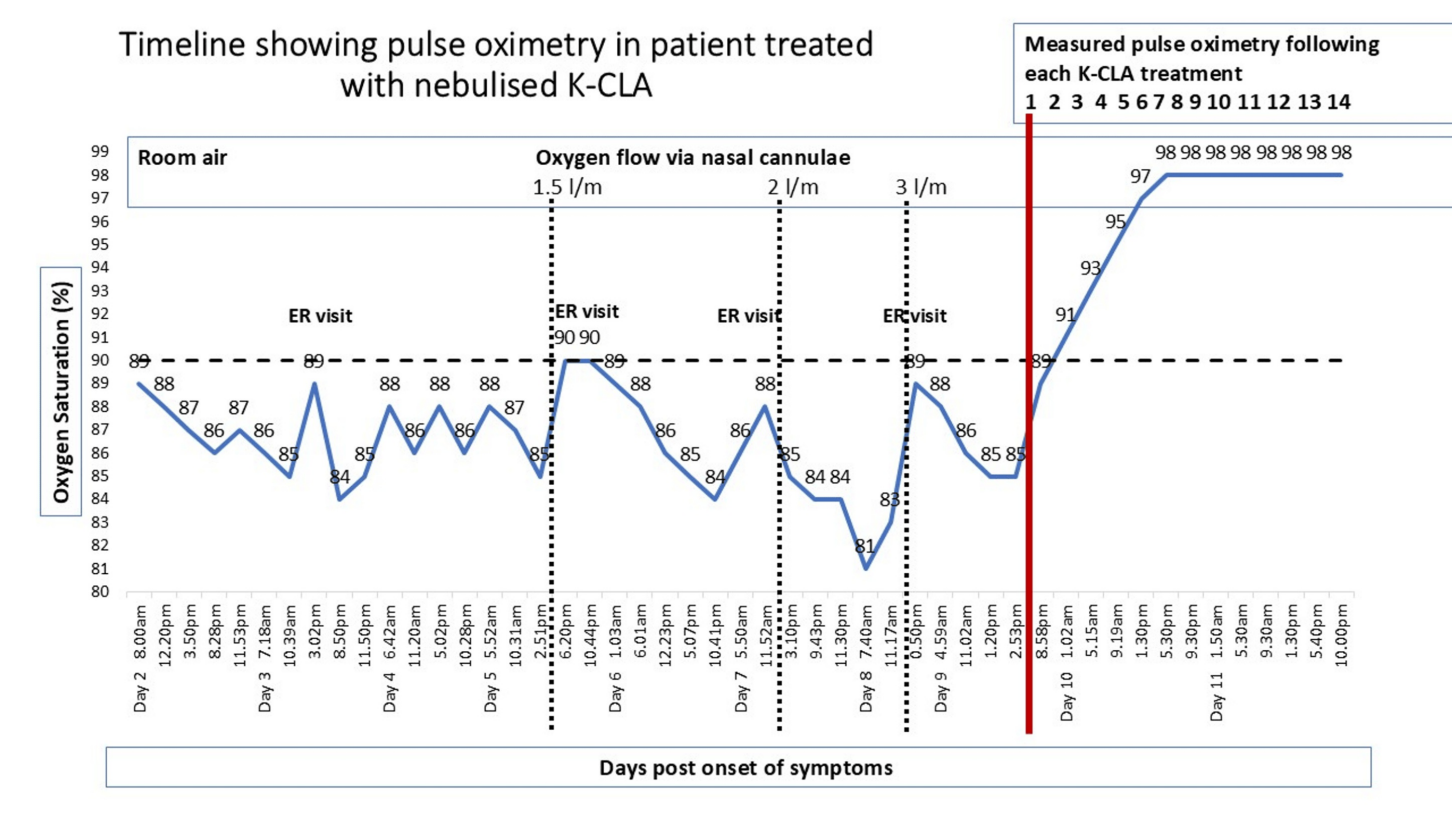
Timeline showing hypoxia improvement following nebulized K-CLA treatment in a 57-year-old male K-CLA - potassium salt of conjugated linoleic acid

His diary demonstrated consistent improvement in oxygen saturation and symptom control following each nebulization treatment. These benefits persisted between each treatment. Pulse oximetry data from his diary is summarized in Figure 2, which shows improvement once nebulized K-CLA was started. Graph of pulse oximetry readings recorded at home by a patient with acute COVID-19 experiencing hypoxia. Included are dates, times, pulse oximetry readings, ER visits, oxygen therapy with dose titration, and each of the first 14 nebulized K-CLA treatments (Figure 2).

A single case of post-COVID-19 syndrome was treated. This patient was a 70-year-old male who presented six months after hospitalization for acute COVID-19 with complaints of persistent dyspnea and fatigue. He had a history of obesity (BMI of 35), gastroesophageal reflux disease, hyperlipidemia, and hypertension. He was admitted to the hospital from day 11 through day 21 of his illness when he was treated with remdesivir and oxygen. He was discharged on four L/m of oxygen via nasal canulae. On day 173, he was weaned off oxygen but continued to use oxygen as needed for activities. 

He started treatment using nebulized K-CLA on day 192. By day 195, he coughed up a large amount of mucus, and his discolored phlegm production subsequently resolved. By day 199, seven days after starting nebulized K-CLA, during his outpatient follow-up visit, he reported that his oxygen saturation while walking up the same incline improved from between 87-89% up to 91%. He no longer needed to use his supplemental oxygen during peak exercise. He also described improvement in fatigue and the need for less sleep. 

## Discussion

A case report of two high-risk patients treated with nebulized K-CLA was previously reported and published [13]. This study provides a larger cohort of patients supporting the human safety data of K-CLA administered by nebulization. Of the 57 subjects treated, three discontinued therapy because of transient side effects. There are no recognized long-term side effects from this study or prior studies.

K-CLA, a conjugated linoleic acid derivative, is considered generally recognized as safe by the FDA for certain food uses. Clinical safety at doses by mouth up to approximately 3-6 g/day has been demonstrated in several trials to be safe without adverse effects [16-17]. However, a meta-analysis found that ≥3.5 g/day of oral CLA over ~six months may raise lipoprotein(a), a cardiovascular risk marker [18]. Yet, the dose of nebulized K-CLA is significantly less (1.96 mg), which is a tiny fraction of 3-6 g/day. Additional lipodystrophy has been reported in a specific CLA isomer at these higher doses, which may impact fatty liver and insulin sensitivity [19]. This was with the CLA isomer trans-10, cis-12, which was not used in this study, and at much higher magnitudes of dosing in oral form.

This retrospective series observed a favorable safety profile for nebulized K-CLA. Anecdotal reports and trends in this cohort suggest it may be associated with reduced symptom severity and duration, particularly when initiated early in the illness. This benefit is thought to be because of the K-CLA anti-SARS-CoV-2 activity. 

In vitro K-CLA exhibits a broad spectrum of antimicrobial properties, and early in the pandemic, it was studied at the Institute of Antiviral Research (Utah State). This showed activity against SARS-CoV-2 at low concentrations, specifically 4.4 mMol, providing the same viral kill rate as 63% ethanol [13], which is the gold standard. 

Linoleic acid, which is closely related to K-CLA, has been shown to bind a hydrophobic pocket in SARS-CoV-2 spike protein, stabilizing it in a closed, noninfectious formation, thereby preventing attachment to the ACE2 receptor and preventing entry into the host cell [11-12]. This mechanism of action is yet to be studied in K-CLA. 

The CLA broad-spectrum antimicrobial mechanism of action may likely be due to its surfactant disruption effect on lipid-based cellular membrane [20-22]. Other unknown virucidal mechanisms are likely, since in vitro viral kill of SARS-CoV-2 is present at low concentrations. 

In a very small, uncontrolled subset of patients (n=7) treated for exposure or asymptomatic infection, none reported progression to symptomatic disease. While intriguing, this observation cannot establish preventative efficacy and requires validation in a controlled trial.

Nebulized K-CLA was also observed to provide improved ventilation, which was supported by improved patient reporting of dyspnea and pulse oximetry in most treated subjects. It should be noted, though, that COVID-19-related hypoxia is multifactorial in origin [23]. 

In this reported patient cohort, 25 patients experienced hypoxia during their illness. Of these, 18 experienced improvements in dyspnea and oxygen saturation after nebulized K-CLA therapy. The observed improvements in dyspnea and oxygenation in some hypoxic patients may be explained by K-CLA's putative surfactant and mucolytic properties, which could theoretically aid in clearing the tenacious mucus characteristic of COVID-19 pneumonia [24].

It is known that the mucus production with COVID-19 can contribute to hypoxia [25-26]. It has also been suggested that surfactant-based prophylaxis or treatment may provide a beneficial approach in COVID-19, as it has already shown benefits in respiratory viral infections [27].

According to Banerjee et al., "pulmonary surfactant is known to stabilize small alveoli and prevent them from collapsing during expiration. However, apart from alveoli, surfactant also lines the narrow conducting airways of the tracheobronchial tree." They suggest exogenous surfactant formulations appear to be beneficial in chronic obstructive airway diseases [28]. Going along with this, it has been reported that the impedance of mucus clearance is a risk factor for respiratory infections [29].

Given the challenge of antibiotic resistance, K-CLA's broad-spectrum antimicrobial, paired with its surfactant action, provides the potential for a new and novel addition to treatment. CLA provides systemic effects that may also be important in clinical outcomes. There have been multiple health claims that need further study. 

A new pandemic is always a potential threat. A 2021 statistical analysis of over 400 years provides an estimated risk of an extreme pandemic of two percent per year. This risk is predicted to increase threefold over the next few decades [30]. Thus, a broad-spectrum antimicrobial with surfactant properties like K-CLA might be a useful tool. 

While nebulized K-CLA appears to be safe, its potential benefits should be interpreted with caution. Although the findings from this case series are encouraging, additional research is needed to confirm its effectiveness. Given its favorable safety profile, a controlled clinical trial would be an appropriate next step to more rigorously evaluate its efficacy. 

Limitations of this study include its retrospective chart-review design, which relied on previously documented records that may contain incomplete data. There is potential for documentation bias, which could skew the results. Additionally, not all confounding variables were captured. Finally, this study cannot establish clinical benefit; however, it does provide preliminary data on safety.

Potential applications for nebulized K-CLA are broad. This includes prevention and treatment of viral respiratory infections, including a potential treatment for the next respiratory pandemic before specific treatments or vaccinations are available. Given the broad-spectrum antimicrobial effect and surfactant action of K-CLA, nebulized K-CLA may provide novel benefits in lung diseases associated with airway mucus retention and infection. This might include asthma, chronic bronchitis, chronic obstructive pulmonary disease (COPD), bronchiectasis, mycobacterium avium complex (MAC), tuberculosis, including extensively drug-resistant tuberculosis (XDR-TB), and cystic fibrosis. The surfactant action of nebulized K-CLA may also provide benefits in acute lung injury scenarios where surfactant dysfunction plays a role, such as post-obstructive pulmonary edema (POPE). Of course, this would need to be confirmed.

## Conclusions

This retrospective case series provides important initial human safety data for nebulized K-CLA, demonstrating it is generally well-tolerated. Three out of 57 patients discontinued treatment because of temporary side effects. The observed clinical outcomes are hypothesis-generating and underscore the necessity of prospective, randomized controlled trials to rigorously evaluate any potential therapeutic or prophylactic effectiveness.
